# Lattice-Based Model of Ductal Carcinoma *In Situ* Suggests Rules for Breast Cancer Progression to an Invasive State

**DOI:** 10.1371/journal.pcbi.1003997

**Published:** 2014-12-04

**Authors:** Eline Boghaert, Derek C. Radisky, Celeste M. Nelson

**Affiliations:** 1 Department of Chemical and Biological Engineering, Princeton University, Princeton, New Jersey, United States of America; 2 Department of Cancer Biology, Mayo Clinic, Jacksonville, Florida, United States of America; 3 Department of Molecular Biology, Princeton University, Princeton, New Jersey, United States of America; Johns Hopkins University, United States of America

## Abstract

Ductal carcinoma *in situ* (DCIS) is a heterogeneous group of non-invasive lesions of the breast that result from abnormal proliferation of mammary epithelial cells. Pathologists characterize DCIS by four tissue morphologies (micropapillary, cribriform, solid, and comedo), but the underlying mechanisms that distinguish the development and progression of these morphologies are not well understood. Here we explored the conditions leading to the emergence of the different morphologies of DCIS using a two-dimensional multi-cell lattice-based model that incorporates cell proliferation, apoptosis, necrosis, adhesion, and contractility. We found that the relative rates of cell proliferation and apoptosis governed which of the four morphologies emerged. High proliferation and low apoptosis favored the emergence of solid and comedo morphologies. In contrast, low proliferation and high apoptosis led to the micropapillary morphology, whereas high proliferation and high apoptosis led to the cribriform morphology. The natural progression between morphologies cannot be investigated *in vivo* since lesions are usually surgically removed upon detection; however, our model suggests probable transitions between these morphologies during breast cancer progression. Importantly, cribriform and comedo appear to be the ultimate morphologies of DCIS. Motivated by previous experimental studies demonstrating that tumor cells behave differently depending on where they are located within the mammary duct *in vivo* or in engineered tissues, we examined the effects of tissue geometry on the progression of DCIS. In agreement with our previous experimental work, we found that cells are more likely to invade from the end of ducts and that this preferential invasion is regulated by cell adhesion and contractility. This model provides additional insight into tumor cell behavior and allows the exploration of phenotypic transitions not easily monitored *in vivo*.

## Author Summary

Breast cancer is a complex disease that affects women worldwide. One heterogeneous group of lesions, ductal carcinoma *in situ* (DCIS), often begins as a nonmalignant disease but can readily progress if left untreated. The progression of this disease is not well understood because DCIS is typically removed upon detection. Therefore, computational models might help predict whether DCIS will remain nonmalignant or progress towards invasive ductal carcinoma. Here we used a multi-cell lattice-based model to explore the relative effects of cell proliferation, death, division axis, adhesion and contractility on the development and progression of DCIS. We also examined the emergence and progression of DCIS in physiologically relevant geometries of the mammary duct. Our model suggests several plausible progressions between morphologies of DCIS, and predicts that some regions of a duct are preferential for tumor cell invasion.

## Introduction

### Ductal carcinoma in situ (DCIS)

**Figure 1:**
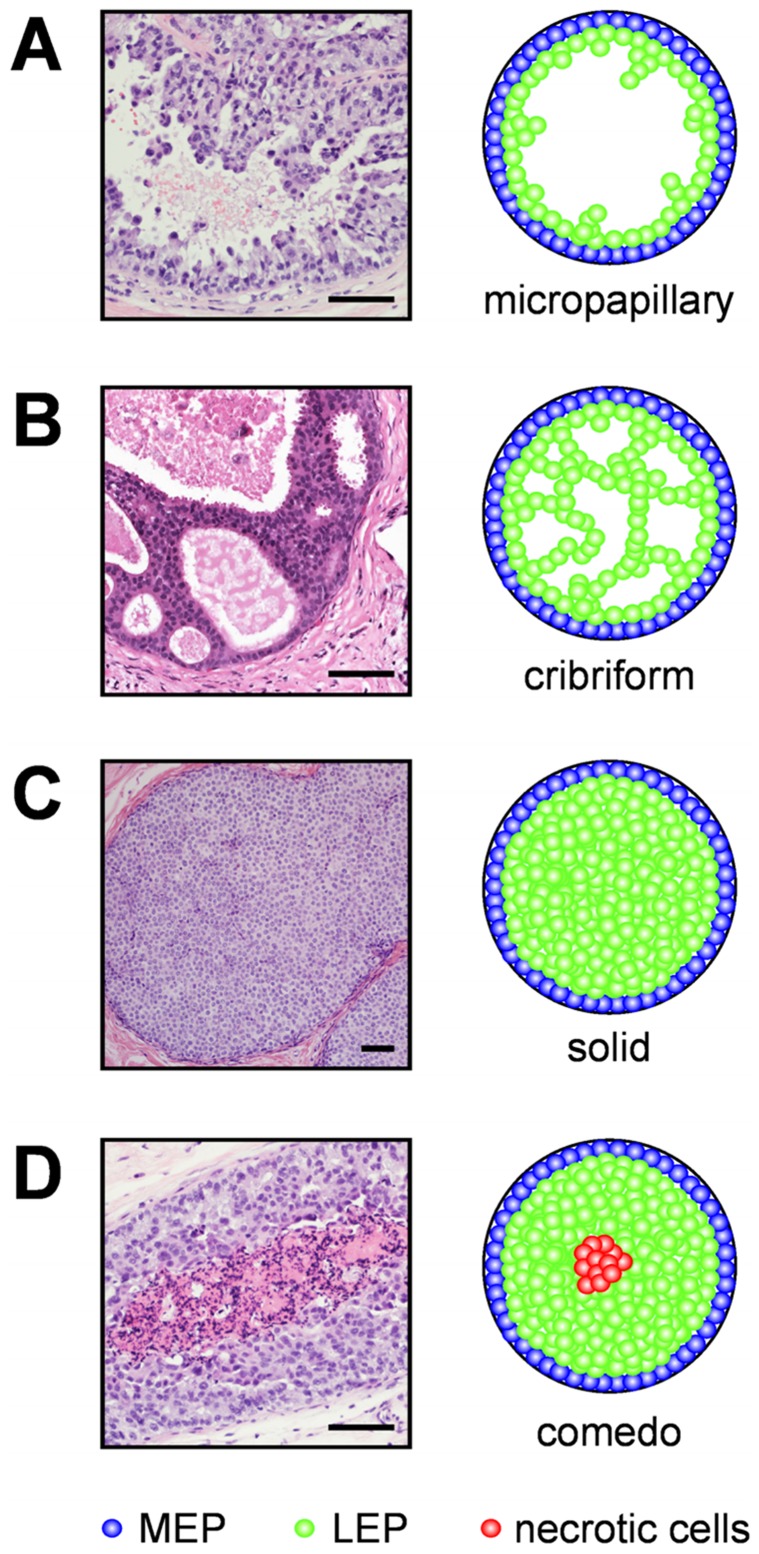
DCIS morphologies. Shown are histology sections (left) and schematic representations (right). (**A**) Micropapillary tumors contain additional epithelial cells within the lumen. (**B**) Cribriform tumors are characterized by ducts filled with cells that form multiple lumena. (**C**) Solid tumors have completely filled ducts. (**D**) Comedo tumors are solid with a necrotic core resulting from nutrient insufficiency [6], [9], [10]. Basement membrane is shown in black, myoepithelial (MEP) cells in blue, luminal epithelial (LEP) cells in green, and necrotic cells in red. Scale bars, 100 µm.

The mammary gland is a highly organized, branched ductal network of luminal epithelial cells surrounded by myoepithelium and basement membrane embedded in stroma [1], [2]. Reciprocal signaling between the cells and their surrounding microenvironment maintains the organization and function of the mammary epithelium. Disruption of these cues and the resulting architecture leads to ductal carcinoma *in situ* (DCIS) and invasive ductal carcinoma (IDC) [1]–[3]. DCIS is defined as increased proliferation of ductal epithelial cells in the absence of basement membrane degradation [4]–[6]. Whereas DCIS is not life-threatening, some of these lesions may progress to IDC if left untreated [7], [8]. Pathologists classify DCIS by four morphologies: micropapillary, cribriform, solid, and comedo. Micropapillary tumors contain additional epithelial cells within the lumen of the duct (**Fig. 1A**). Cribriform tumors are characterized by ducts filled with cells that form multiple lumena (**Fig. 1B**). Solid tumors have completely filled ducts (**Fig. 1C**). Comedo tumors are solid with a necrotic core resulting from nutrient insufficiency (**Fig. 1D**) [6], . Of these four morphologies, comedo lesions have the greatest risk for recurrence after breast-conserving surgery [11]. Due to the increased use of mammographic screening, the number of observed incidences of DCIS has increased dramatically, by 500% and 290% between 1983 and 2003 for women over 50 and under 50, respectively [12]. DCIS currently accounts for ∼20% of all breast cancers diagnosed in the U.S. [8].

It remains unclear how DCIS evolves into invasive breast cancer. In most cases, DCIS is detected by mammography in an otherwise asymptomatic patient; the lesions are then removed surgically after detection and so the natural history of the lesion cannot be monitored *in vivo* [5]. Of these lesions, invasive carcinomas develop more frequently in patients treated with biopsy alone than in patients who receive lumpectomy followed by radiation treatment [13], [14], highlighting the need for prognostic stratification and diligent monitoring. Published clinical studies show that 14–53% of DCIS originally misdiagnosed as benign breast disease later develops into invasive breast cancer [8]. Furthermore, DCIS and invasive cancers often have the same morphological appearance and genetic profile, suggesting that they originate from the same source, and DCIS and invasive morphologies are often present in the same lesion [8], [15]. Computational models may help to predict which conditions lead to the development of the various morphologies of DCIS, and perhaps suggest plausible mechanisms by which DCIS evolves to invasive carcinoma.

### Microenvironment and tumor phenotype

Recent work has emphasized the profound effects that the cellular, chemical, and physical properties of the tumor microenvironment can have on tumor progression [16]–[23]. In some instances the microenvironment provides a tumor-suppressive role, as autopsies have revealed that 20% of young and middle-aged women have clinically occult breast tumors [24], whereas in other instances tumors readily progress to malignant carcinoma. Previously, we found that the mechanical properties of the host epithelium play a critical role in establishing or suppressing a tumorigenic phenotype in cells with a tumorigenic genotype. We incorporated human breast tumor cells into engineered tissue mimetics comprised of non-malignant host mammary epithelial cells, and observed that the tumor cells proliferated or invaded *only* when they were located at the ends of these tissues [25]. These sites of tumor cell invasion corresponded to regions of high endogenous mechanical stress. Furthermore, this dependence of tumor cell phenotype on location within the tissue could be modulated by altering the contractility, and thus the mechanical stress profile, of the host epithelium [25]. These location-dependent differences in tumor cell behavior strengthen the importance of studying tumorigenesis in the context of the tissue and its mechanical microenvironment [26].

Modeling DCIS within a sphere or within the circular cross-section of a single duct fails to capture these architecture-dependent variations in the microenvironment. More than 90% of all human mammary carcinomas originate in the epithelial ducts rather than the surrounding connective tissue [27], and the majority of these arise from the terminal ductal lobular unit [28], suggesting that the microenvironment around the terminal ends of the ducts is more supportive (or less suppressive) of tumor formation. Recent experimental work has revealed that tissue geometry establishes varying levels of mechanical stress and morphogen concentrations within mammary ducts [29]–[31]. These variations may establish regions that are preferential for tumor cell proliferation and invasion [25]. In addition, patients diagnosed with DCIS have frequently been found to have lesions with heterogeneous morphology [32], suggesting that a radial cross-section of a single duct *in silico* cannot accurately predict tumor formation. In this study we begin by exploring the behavior of DCIS in a two-dimensional circular cross-section of a duct, thus allowing us to compare the results of our model to those of previously published studies. We then expand our model and vary the geometry of the tissue to examine regional differences in tumor cell behavior.

### Domain-based modeling of tumor phenotype

The computational model presented here was developed using the cellular Potts model (CPM) implemented through the CompuCell3D modeling platform. The CPM is a multi-cell lattice-based model that uses fairly few parameters to describe effective interactions and constraint energies within biological systems [33], [34]. CPM has been used to study both normal developmental processes including morphogenesis of the embryonic limb bud [35] as well as pathological processes associated with tumor behavior [36]–[38]. Andasari et. al. developed a multi-scale model to examine cancer growth and invasion resulting from intracellular dynamics of E-cadherin and β-catenin and found that lowering cell adhesion caused increased cell invasion [39]. Steinkamp et. al. used both mouse tumor models and a computational model to better understand ovarian tumor growth and morphology due to oxygen availability and tumor cell adhesion. These authors found that strong homotypic adhesion and weak heterotypic adhesion are required for cancer cells to form spheroid aggregates. Furthermore, variations in cell adhesion led to the establishment of different tissue microenvironments; cancer cells invaded preferentially into the microenvironment of the mesentery, omentum and spleen and did not invade into that of the stomach and small intestine [40]. Here we used CPM to explore the conditions that lead to the development of the different morphologies. We observed several plausible progressions between these four morphologies of DCIS. We also examined variations in phenotype that result from geometric features of mammary epithelial ducts, and observed that some regions are preferential for tumor cell invasion and that this invasion can be modulated by tuning cell adhesion and contractility.

## Model

### Cellular Potts model

We used Glazier and Graner's cellular Potts model (CPM), implemented through the open-source simulation environment CompuCell3D (http://www.compucell3d.org), to create a two-dimensional (2D) model of DCIS. In this framework, each agent is a domain of pixels given a unique index, *σ* representing a cell compartment, cell, or tissue.As the model progresses, agents attempt to extend their boundary. One Monte Carlo step (MCS) is defined as one attempt for each pixel in the model to alter its location. The success of these attempts is given by the probability:
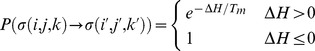
where *ΔH* is the change in effective energy, which we describe below, *T_m_* is the parameter “temperature,” corresponding to the intrinsic agent motility, and the indices (*i*, *j*, *k*) specify lattice sites (pixels) [33], [34], [41].

The change in effective energy is defined by three main terms as shown below:
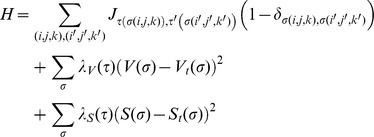
where τ denotes the type of agent. The first term describes the surface adhesion energy between agents and their neighbors, essentially establishing the degree to which agents sort from one another. The second term defines the volume and the compressibility of the agent. The difference between the volume (*V*) and the target volume (*V_t_*) is multiplied by a parameter that describes the stiffness of the agent (*λ_V_*). The third term of the Hamiltonian represents the elasticity of the agent membrane, where *S* is the surface area and *S_t_* is the target surface area [33], [34], [41]. An in-depth review of the Glazier-Graner CPM model framework is given in Ref [34]. Parameters used here are summarized in **Table 1** and discussed below.

**Table 1:** Model parameters and variables.

***Adhesion Parameters***	
*J_LEP,LEP_*	−20*
*J_LEP,MEP_*	−10*
*J_MEP,MEP_*	−5*
***Volume Parameters***	
*V_t,LEP_*	78.5
*λ_V,LEP_*	5*
*V_t,MEP_*	78.5
*λ_V,MEP_*	10*
***Surface Parameters***	
*S_t,LEP_*	31.4
*λ_S,LEP_*	1
*S_t,MEP_*	31.4
*λ_S,MEP_*	1
***Elasticity Parameters***	
*L_LEP,LEP_*	8
*λ_LEP,LEP_*	50*
*L_LEP,MEP_*	8
*λ_LEP,MEP_*	5*
*L_MEP,MEP_*	5
*λ_MEP,MEP_*	50*
***Proliferation***	
time between mitotic events	32–90 MCS*
***Apoptosis***	
probability	0–1%*

Values denoted with a * vary during implementation of the model as described in the text.

#### Model geometry

Our initial layout mimicked the cross-sectional geometry of a normal mammary duct and contained a layer of 50 luminal epithelial cells (LEP) surrounded by a layer of myoepithelial cells (MEP), each with a diameter of 10 µm [42], [43]. Our simulations used a scale of 1 pixel = 1 µm. Since the model uses a square lattice, the cells in each simulation began as squares with a width of 10 pixels. The target volume and surface area were chosen so that each cell was circular with a diameter of 10 µm; we found that the cells became rounded within 10 MCS. In addition to a circular geometry, we examined cylindrical and bifurcating ductal geometries, as described in the Results section.

The λ volume parameter corresponds to the elasticity of a material. Atomic force microscopy (AFM) measurements have shown that MEP have a Young's modulus that is twice that of LEP [44]. Consistent with these observations, values for *λ_V,MEP_* and *λ_V,LEP_* were chosen in a 2∶1 ratio. Previous studies have used *λ_V_* = 3 for Ecell ∼0.5 kPa [37]; since *E*_MEP_ ∼1.5 kPa [44], we chose values of 10 and 5. Similarly, the *λ* surface parameter was set to 1 so that the model is not too rigid, but cells do not become fragmented.

### Cell adhesion

Mechanical links between cells were established through the adhesion coefficient, which is defined as the adhesion energy per unit contact area and lowers the effective energy of the system when bonds form between cells. A hierarchy of surface adhesion coefficients leads to cell sorting [45]. Experimentally, it has been shown that the physiological organization of LEP surrounded by a layer of MEP results from differential expression of E-cadherin [44]. We modeled strong cellular adhesion using negative values for the adhesion coefficients and modeled the differential expression of E-cadherin by setting *J_LEP,LEP_*<*J_MEP,LEP_*<*J_MEP,MEP_*. Adhesion coefficients for agent pairs that include culture medium or necrotic cells were set to zero, since cells that undergo necrosis continue to occupy space as debris, but no longer bind with other cells. This is equivalent to placing the cells in a very deformable stroma, which was not included *per se* in the current model.

Additionally, cell attraction and repulsion were modeled using the focal point plasticity (FPP) plugin, which creates a link between the centers of mass of neighboring agents. The energy term is defined as 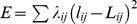, where *L_ij_* is the target distance between the agents' centers of mass, *l_ij_* is the actual distance, and *λ_ij_* is equivalent to a spring constant. We established cell polarity by allowing epithelial cells to form links with only two neighbors [10]. We set the *λ_ij_* parameter for homotypic cellular interactions to be 10-fold greater than the *λ_ij_* parameter for heterotypic cellular interactions [10]. In order to mimic cell contraction, the target length between cells was set to be less than the resting length. For the more contractile MEP, we set the target length at 5 pixels while for LEP we set the target length at 8 pixels. When FPP parameters are set too high, cells achieve their target length by becoming fragmented. FPP parameters for agent pairs that include culture medium or necrotic cells were set to zero.

#### Cell proliferation

**Figure 2:**
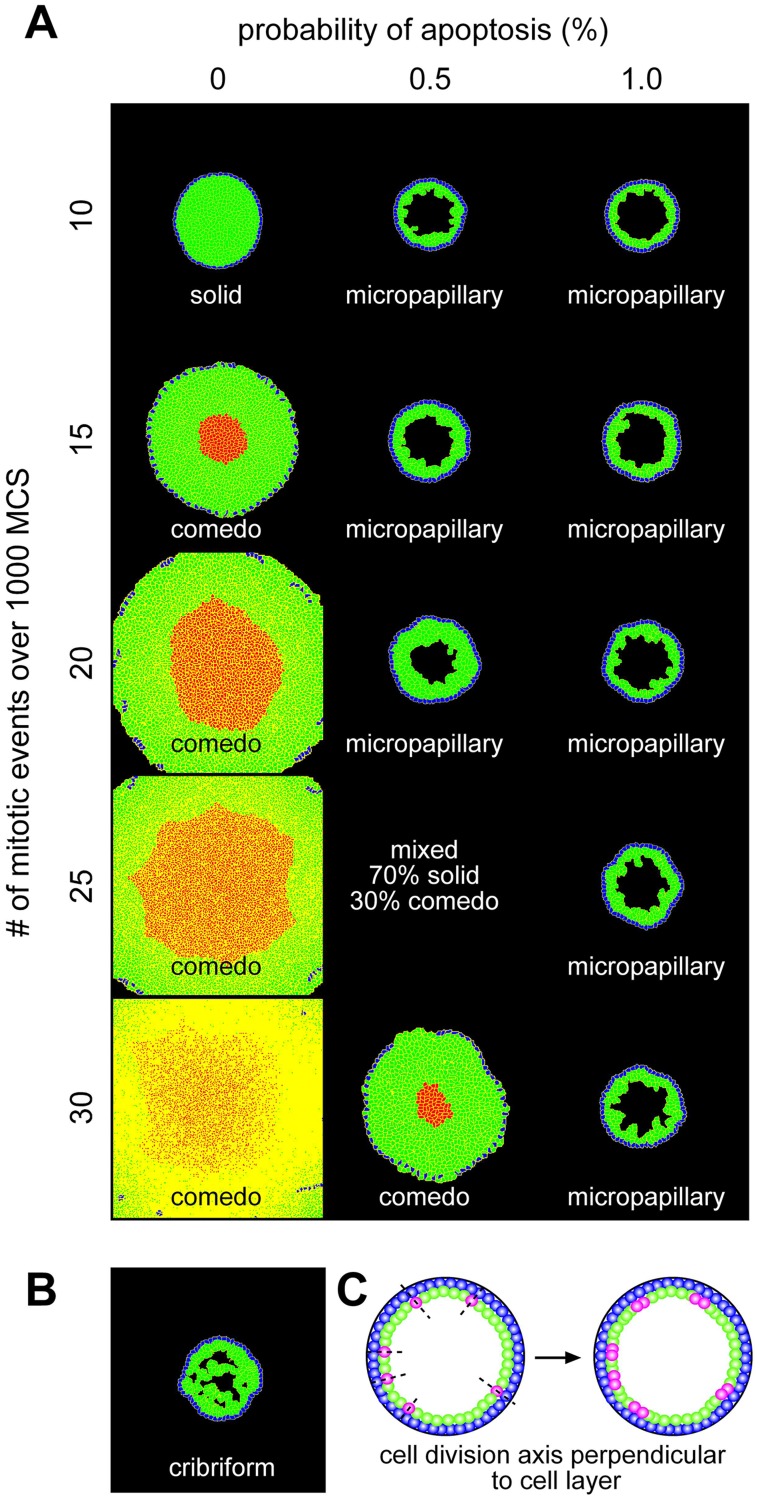
Generation of morphologies based on number of mitotic events and probability of apoptosis. (**A**) Varying the probability of apoptosis and the mitosis frequency, we observe the emergence of solid and comedo morphologies at high proliferation rates with low apoptosis and micropapillary morphology at low proliferation rates with high apoptosis. (**B**) The cribriform morphology emerged occasionally, but not consistently for any of these conditions. Image shown from 1% apoptosis and 25 mitotic events. (**C**) Schematic of cell division when the division axis is specified to be perpendicular to the epithelial cell layer. Cells shown in pink undergo cell division.

In normal ducts, luminal epithelial cells are polarized and enter a state of growth arrest [46]. Two explanations for this phenomenon have been proposed and validated computationally. In one, normal luminal epithelial cells lose the ability to proliferate when they form tight junctions with their neighbors [47], [48]. In the other, cells continue to proliferate but progeny that enter the lumen subsequently undergo apoptosis [49]. These normal control mechanisms are subverted during DCIS [5]. Here, one quarter of the luminal epithelial cells were chosen randomly and set to proliferate at a given time step, with the axis of cell division perpendicular to the epithelial cell layer (**Fig. 2C**). In the Results section we discuss the effect of changing the orientation of cell division. We varied the frequency of cell proliferation during the simulation from 10–30 mitotic divisions during 1000 MCS to examine the effects of proliferation on the emergence and progression of DCIS. It has been suggested that cells proliferate more rapidly when located at the outer rim of the lesion [50], [51] which would be expected to alter the pattern of mechanical stresses within the duct; such proliferation patterns have not been widely documented and were not modeled here.

### Cell death

We considered two types of cell death, apoptosis and necrosis. During normal development a luminal epithelial cell undergoes apoptosis when it experiences mechanical stress from being overcrowded, and is then extruded from the tissue [52], [53]. In our model, we first checked whether a cell was overcrowded by counting the number of neighbors that were within 2.5 cell diameters. If there were 10 cells in this vicinity, the luminal epithelial cell was specified as overcrowded and apoptosis was inscribed with a given probability ranging from 0 to 1%. When a cell underwent apoptosis it was removed from the simulation. Necrosis results from a lack of nutrient availability. Since DCIS does not involve angiogenesis into the duct [54], [55], the closest nutrient source would be immediately adjacent to the duct itself. Here we specified that a cell would undergo necrosis when it was ten or more cell diameters away from the myoepithelial layer (≥100 µm), a distance roughly equal to the diffusion limit of oxygen. When a cell became necrotic, it no longer interacted with other cells and all cell-cell adhesion parameters were set to zero; however, the necrotic cell continued to occupy space.

## Results

### Morphology of DCIS depends on the relative rates of proliferation and apoptosis

We first explored the changes in mammary ductal morphology that resulted from altering rates of cell proliferation and apoptosis (**Fig. 2**). To determine the predominant morphology, we ran each simulation at least 20 times. If 80% or more of these simulations led to the same morphology, we classified it as such. Otherwise, we concluded that the conditions led to a mixed morphology. We found that the micropapillary morphology emerged under most conditions, with notable exceptions. In the absence of apoptosis (**Fig. 2A**, left column), we observed both the solid and comedo morphology, depending on the frequency of cell division. For 10 mitotic events, the LEP completely filled the lumen. As the cells divided they imposed an outward force on the walls of the duct causing the duct to expand. When there were 15 or more mitotic events, the contractility of the myoepithelial layer could not balance this outward force, and we observed gaps in the MEP layer as well as the presence of a necrotic core. Notably, even though the LEP breached through the MEP layer, the duct still maintained a circular cross-sectional morphology; such gaps in the MEP layer have been observed in histological sections of human breast tumors diagnosed as DCIS [56], [57]. Similarly, when the probability of apoptosis was set to 0.5% (**Fig. 2A**, center column), solid and comedo morphologies were established under conditions of high proliferation (25 or 30 mitotic events) whereas the micropapillary morphology emerged under conditions of low proliferation (20 or fewer mitotic events). When the probability of apoptosis was increased to 1% (**Fig. 2A**, right column), micropapillary morphologies were established. The cribriform morphology was observed in a few simulations; however, it was not the predominant morphology under any of these conditions. For example, with 1% probability of apoptosis and 25 mitotic events, one in twenty simulations resulted in a cribriform morphology (**Fig. 2B**). This result was surprising, given that 16–20% of all cases of DCIS have been described as cribriform in morphology [58], [59]. These data suggest that the morphology of DCIS may depend on the balance between cell division and apoptosis, which is supported by collapsing the data into a single ratio of proliferation to apoptosis (**S1 Figure**). The lumen fills and eventually becomes necrotic when this balance is shifted toward proliferation. The lumen remains patent, albeit abnormal, when this balance is shifted toward apoptosis. The low incidence of cribriform morphology observed in these simulations suggests that additional cellular behaviors are required to generate this architecture.

### Development of multiple lumena depends on cell division axis

**Figure 3:**
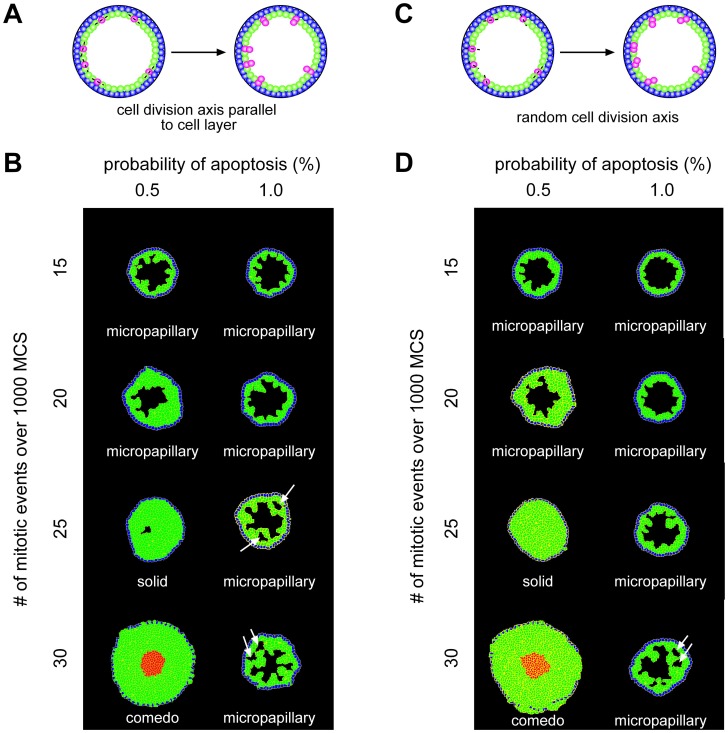
Varying cell division axis leads to increased development of multiple lumena. Schematic of cell division with the cell division axis specified to be parallel to the epithelial cell layer (**A**) or random (**C**). Cells shown in pink undergo cell division. Varying the probability of apoptosis and mitosis frequency, we observe an increased emergence of multiple lumena when the cell division axis is parallel to the epithelial cell layer (**B**) or chosen randomly (**D**).

As described above, there are two possible mechanisms by which cells in a normal duct can undergo growth arrest. In one, normal epithelial cells lose the ability to proliferate when they form tight junctions with their neighbors [47], [48]. In the other, cells continue to proliferate but any daughter progeny that occupy the lumen immediately undergo apoptosis [49]. Thus, we next explored how the axis of cell division affects the morphology of the simulated duct. In the simulations described above (**Fig. 2**), we had specified the axis of cell division to be perpendicular to the epithelial cell layer (**Fig. 2C**); next we investigated the effects of cell divisions that introduced progeny into the lumen which were protected from undergoing apoptosis (0% probability; **Fig. 3A, B**), or allowing the cells to undergo random cell division thereby resulting in a loss of tissue polarity (**Fig. 3C, D**). Regardless of the axis of cell division, solid and comedo morphologies were established under combinations of high proliferation (25 or more mitotic events) and low apoptosis (0.5% probability). When the cell division axis was random or such that daughter cells were placed into the lumen, the duct appeared to expand slightly more than when the division axis was perpendicular to the epithelial cell layer. The former caused a small lumen to appear in conditions that otherwise led to a solid morphology (compare **Fig. 2A** solid morphology to **Fig. 3B** solid morphology). For example, with 0.5% apoptosis and 25 mitotic events, the duct became almost completely filled with LEP; however, in half of the simulations a very small lumen remained.

Although the cribriform morphology did not arise as the predominant morphology under any of these conditions, we found many ducts containing small lumena, particularly when the axis of cell division was parallel to the epithelial layer (see white arrows in **Fig. 3B and 3D**). These results are consistent with observations that cells maintain apicobasal polarity in cribriform lesions [60]. We did not characterize the duct as cribriform unless cells extended completely across the diameter of the duct (**Fig. 2B**). As the number of mitotic events increased, the number of ducts with cribriform morphology also increased. With 1% apoptosis, the percentage of cribriform ducts increased from 2% to 6% to 18% for 20, 25 and 30 mitotic events respectively. This suggests that the cribriform morphology may occur more readily over a longer time span with more cell divisions. These data again suggest that the morphology of DCIS depends on the balance between cell division and apoptosis.

### Progression of DCIS morphologies

**Figure 4:**
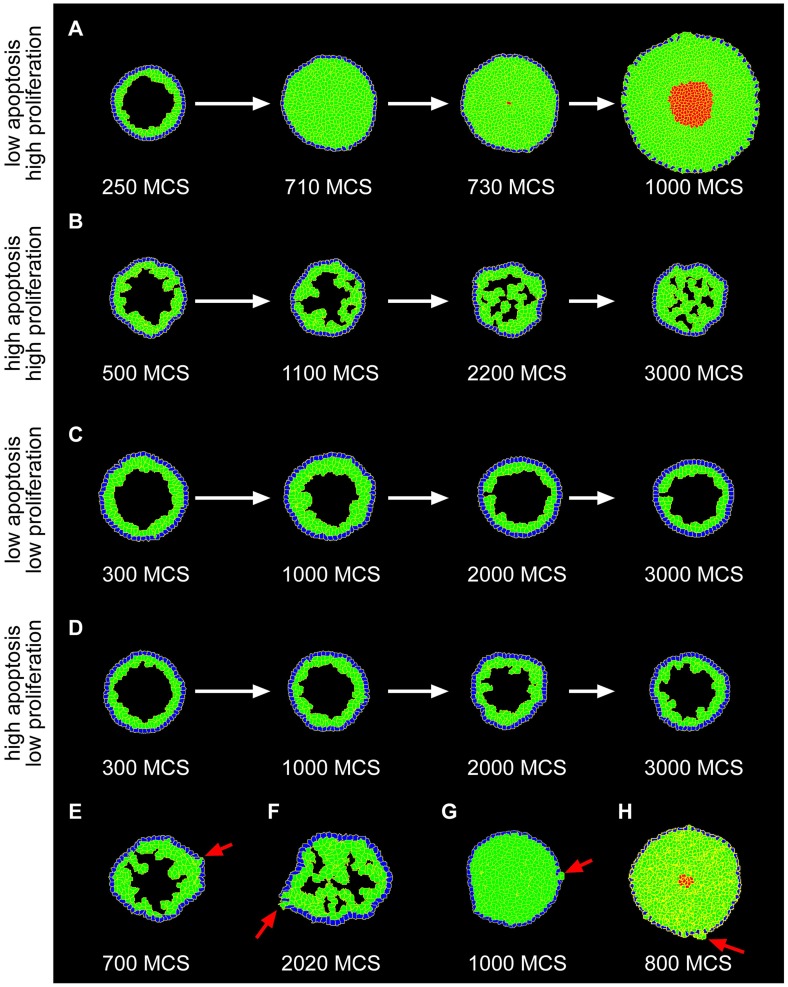
Progression between DCIS morphologies. (**A**) With no apoptosis and high proliferation (mitosis every 65 MCS, 15 mitotic events over 1000 MCS) we observe transitions from micropapillary to solid morphology and from solid to comedo morphology. Cell division axis was specified perpendicular to the epithelial layer. (**B**) With high apoptosis (1% probability) and high proliferation (mitosis every 38 MCS, 25 mitotic events over 1000 MCS) we observe transitions from the micropapillary to the cribriform morphology. Cell division axis was specified parallel to the epithelial layer. (**C**) With low apoptosis (0.5% probability) or (**D**) high apoptosis (1% probability) and low proliferation (mitosis every 65 MCS, 15 mitotic events over 1000 MCS) we observe only the micropapillary morphology. Cell division axis was random. LEP are capable of invading through the MEP layer from (**E**) micropapillary, (**F**) cribriform, (**G**) solid, and (**H**) comedo morphologies. The images shown here were generated under the following conditions: (**E** and **F**) 1% probability of apoptosis, mitosis every 32 MCS (30 mitotic events over 1000 MCS), and cell division axis parallel to the epithelial layer; (**G**) 0.5% probability of apoptosis, mitosis every 32 MCS (30 mitotic events over 1000 MCS), and random cell division axis; (**H**) 0.5% probability of apoptosis, mitosis every 38 MCS (25 mitotic events over 1000 MCS), and cell division axis parallel to the epithelial layer.

Whereas it is difficult to explore the transitions between DCIS morphologies in intact tumors *in vivo*, this is readily achieved *in silico*. Examining intermediate time steps and running simulations for up to 3000 MCS, we observed multiple transitions between morphologies. As LEP accumulated in the lumen, the micropapillary morphology was the first to emerge. In the absence of apoptosis, or at low levels of apoptosis (0.5% probability) with high proliferation, the micropapillary morphology progressed to solid and ultimately to comedo as the force of proliferating cells caused the duct to expand outward (**Fig. 4A**). At higher levels of apoptosis (1% probability) and high levels of proliferation, the micropapillary morphology progressed to cribriform (**Fig. 4B**). With low levels of proliferation, the morphology remained micropapillary (**Fig. 4C, D**). Increasing apoptosis from 0.5% probability to 1% probability did not affect the outcome of these simulations. Notably, high levels of apoptosis or low levels of apoptosis balancing low levels of proliferation caused the duct to remain fairly uniform in size (**Fig. 4B–D**). Given that the cells continued to proliferate, we had anticipated that the lumen would fill completely and the cribriform morphology would ultimately progress to a solid morphology. Surprisingly, however, the morphology remained cribriform even after 3000 MCS under conditions of 1% probability of apoptosis and high proliferation (**Fig. 4B**). This suggests that over longer periods of time, comedo and cribriform may be the ultimate morphological outcomes of DCIS, with apoptosis being the deciding factor. Importantly, we found that LEP were able to break through the MEP layer into the surroundings from any of the four morphologies (**Fig. 4E–H**). For our purposes here we refer to this phenotype as invasion; however, we note that physiological invasion *in vivo* requires deterioration of the basement membrane, which is not included in the present model.

### Asymmetric tissue geometry establishes regions preferential for invasion

**Figure 5:**
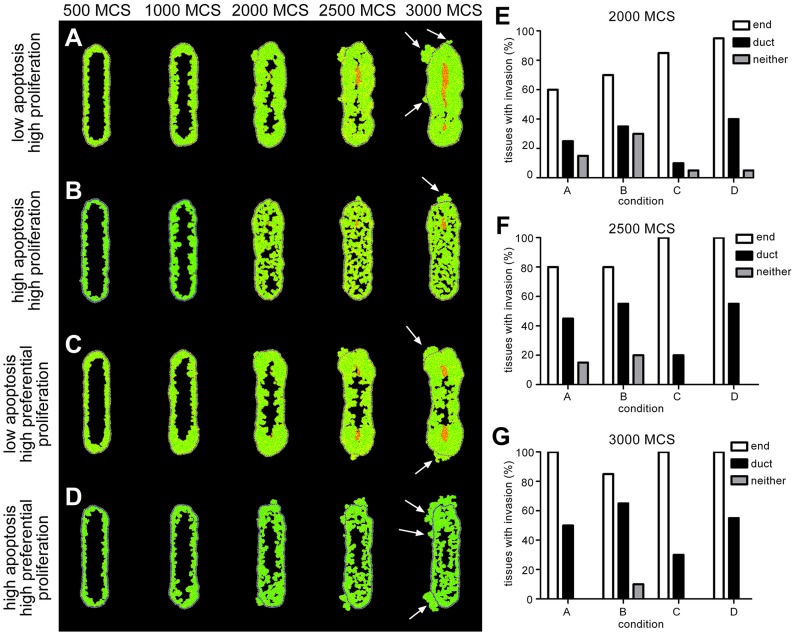
Invasion occurs preferentially at the ends of cylindrical ducts. (**A**) 0.5% probability of apoptosis, mitosis every 48 MCS, and random cell division: morphology begins as micropapillary and develops into cribriform with necrotic cells in the center and some invasion. Eventually the tissue becomes comedo. (**B**) 1% probability of apoptosis, mitosis every 32 MCS, and random cell division: morphology begins as micropapillary and develops into cribriform. As the tissue expands there are some necrotic cells in the duct and some cells break through the myoepithelial layer. (**C**) 0.5% probability of apoptosis, mitosis every 48 MCS, random cell division, and preferential proliferation: morphology begins as micropapillary and develops into cribriform morphology in the duct region with comedo morphology and invasion at the ends. Eventually the entire tissue becomes comedo. (**D**) 1% probability of apoptosis, mitosis every 32 MCS, random cell division, and preferential proliferation: morphology begins as micropapillary and develops into cribriform. As the tissue expands there are some necrotic cells in the duct and some cells break through the MEP layer. Quantification of tissues with invasion at the end and the duct region of each tissue at (**E**) 2000 MCS, (**F**) 2500 MCS and (**G**) 3000 MCS. Simulations were run 20 times each for the parameters described in A–D. Invasion occurs preferentially at the ends of the tissues.

Experiments in culture have revealed that asymmetries in tissue geometry lead to regional differences in endogenous mechanical stress, which result from the concentration of mechanical stresses by otherwise isotropically contracting cells in the tissue [29], [30]. Furthermore, tumor cells have been observed to proliferate and invade preferentially from regions of high mechanical stress both in culture and *in vivo* [25]. We next explored whether tissue geometry affected the morphology that emerged by modeling a cross-section through a cylindrical (ductal) tissue. Throughout the tissue, the morphology of DCIS that emerged appeared to be fairly consistent; however, we observed that cells invaded more frequently from the ends than from the center of the duct (**Fig. 5A, B, E–G**). Previously, we found experimentally that tumor cells proliferate almost twice as frequently when they are located at the ends of ducts engineered in culture [25]. When we included this pattern of proliferation in our model, we observed an increase in the number of tissues in which cells invaded from the ends (**Fig. 5C, D, E–G**). We also noticed some differences in morphology. For example, in the simulations shown in **Fig. 5C**, the duct region of the tissue develops into a cribriform morphology while the end region becomes comedo with invasion. Experimentally, preferential invasion has been attributed to elevated levels of mechanical stress [25], possibly due to mechanical regulation of YAP/TAZ [61]. When cells push and pull on each other within a tissue, varying levels of endogenous mechanical stress will emerge across the tissue due to asymmetries in the tissue geometry [29], [31]. Therefore, we next explored the effect of altering tissue contractility in this model.

### Cell adhesion and tissue contractility control preferential invasion

**Figure 6:**
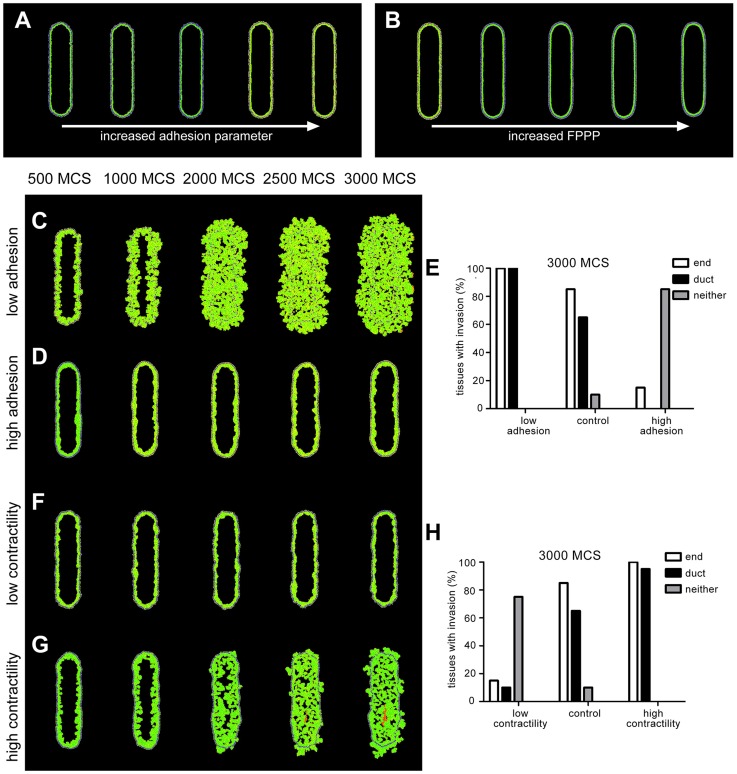
Patterns of cell invasion depend on cell adhesion and contractility. In the absence of proliferation, changing the adhesion parameter and the focal point plasticity parameter does not significantly alter tissue structure. (**A**) Images were generated using *J_LEP,LEP_*, *J_MEP,LEP_*, and *J_MEP,MEP_* values of −2, −1, −0.5; −10, −5, −2.5; −20, −10, −5; −40, −20, −10; and −100, −50, −25 with FPPP set to zero. (**B**) Images were generated using FPP parameters of 5 and 0.5, 25 and 2.5, 50 and 5, 75 and 7.5, and 100 and 10 for homotypic and heterotypic interactions, respectively. With high proliferation (mitosis every 65 MCS) and high apoptosis (1% probability), changing the adhesion parameter and the focal point plasticity parameter affects cell invasion. (**C**) When cell adhesion is decreased cells invade from the entire periphery of the tissue. Adhesion and FPP parameters were all set to 0. (**D**) Increased cell adhesion inhibits invasion. Adhesion parameters were set to −100, −50, and −25 for *J_LEP,LEP_*, *J_MEP,LEP_*, and *J_MEP,MEP_*, respectively. (**E**) Quantification of tissues with invasion at the end and the duct region of each tissue at 3000 MCS. Simulations were run 20 times each for the parameters described in C–D. (**F**) When tissue contractility is decreased by lowering the *λ_v,MEP_* and *λ_v,LEP_* to 2 and 1, respectively and lowering FPPP to 1 and 0.1 for homotypic and heterotypic cell interactions, cell invasion is inhibited. (**G**) Increased tissue contractility increases invasion from the duct regions. *λ_v,MEP_* and *λ_v,LEP_* were set to 50 and 25, respectively and FPP parameters were increased to 100 and 10 for homotypic and heterotypic cell interactions. (**H**) Quantification of tissues with invasion at the end and the duct region of each tissue at 3000 MCS. Simulations were run 20 times each for the parameters described in F–G.

In our model, the ability of cells to adhere to each other is regulated by the cell adhesion and FPP parameters. In the absence of proliferation, changing these parameters did not significantly alter the structure of the tissue (**Fig. 6A, B**). However, as cells proliferated and produced an outward force, the roles of these parameters became more significant. When the value of the cell adhesion parameter was decreased, the cells no longer adhered to each other and invasion was observed around the entire periphery of the tissue (**Fig. 6C, E**). When the value of the cell adhesion parameter was increased, the strength of cell adhesion prevented invasion (**Fig. 6D, E**). Notably, high adhesion caused the morphology to remain micropapillary, whereas low adhesion led to the development of a cribriform morphology (**Fig. 6D, E**).

In addition to controlling cell adhesion, the FPP parameter also modulates tissue contractility. In our model, tissue contractility was approximated by a balance of FPP and *λ_v_* parameters. The FPP parameter connects the centers of each cell with a spring, the target length of which is set to be less than the resting length, thereby creating an attractive pull between two cells. As the cells are pulled together, the *λ_v_* parameter creates an outward push by imposing a penalty when the cell deviates from its target volume. When these two forces are balanced, the result is a tissue in a state of tension (**Fig. 6B**, center tissue). When we lowered these parameters, essentially eliminating contractility from our model, the cells did not invade (**Fig. 6F, H**). We did observe breaks in the myoepithelial layer in most tissues; however, since the LEP cells did not extend past the periphery of the tissue, we did not characterize these breaks as invasion. Increasing the value of these parameters caused cells to invade from the entire periphery of the tissue (**Fig. 6G, H**). These results are in agreement with our previously published experimental results, in which we found that reducing tissue contractility eliminates tumor cell invasion, whereas increasing contractility permits tumor cells to invade from the duct region of the tissue [25]. Again there were notable differences in DCIS morphology. Low contractility caused the morphology to remain micropapillary, whereas high contractility led to the development of a cribriform morphology with necrotic cells in the center of the tissue (**Fig. 6F, G**). The increased accumulation of cells in the interior of the tissue is in agreement with a recent agent-based model that showed that clusters of tumor cells grow faster as the *λ_v_* parameter is increased [62].

### Preferential invasion occurs at the ends of bifurcating ducts

**Figure 7:**
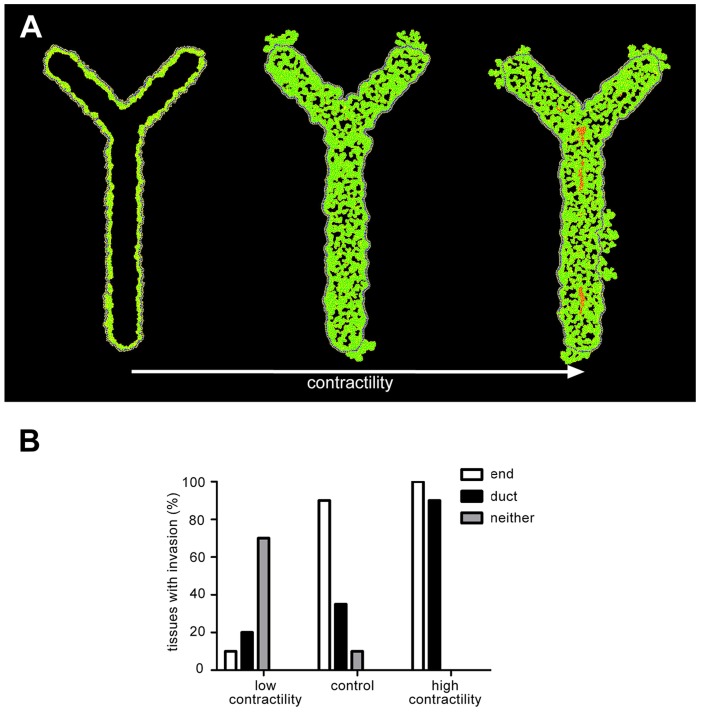
Cells invade preferentially from the ends of bifurcating ducts. (**A**) In control tissues cells invade preferentially from the ends of bifurcating ducts. Decreasing contractility partially inhibits invasion, while increasing contractility causes delocalization of invasion. (**B**) Quantification of tissues with invasion at the end and the duct region of each tissue at 3000 MCS. Simulations were run with high proliferation (mitosis every 65 MCS) and high apoptosis (1% probability), 20 times for each of the parameters described in **Fig. 6F–G**.

We previously used a transgenic mouse expressing an inducible form of the kRas oncogene under control of the mouse mammary tumor virus (MMTV) promoter to observe tumor development *in vivo* in the post-pubertal mammary gland. These studies revealed that tumors form more frequently at the ends of the complex network of epithelial ducts in adult mice [25]. We thus expanded our computational model to examine tumor growth in a bifurcating duct, and observed that tumor cells invaded more often from the ends of the bifurcating duct. Using the same parameters for low and high contractility described above and presented in **Fig. 6**, we explored the effect of altering tissue contractility. Low contractility caused the morphology to remain micropapillary, whereas high contractility led to the development of a cribriform morphology with necrotic cells in the center of the tissue (**Fig. 7A**). Again we found that invasion was reduced by decreasing contractility and delocalized by increasing contractility (**Fig. 7**). Agreement between these *in vivo* and computational results suggests that this model could be expanded to predict tumor cell behavior in increasingly complex physiologically relevant geometries.

## Discussion

In order to better understand the development of breast cancer, it is beneficial to investigate the mechanisms by which the ductal architecture of the normal mammary gland is established and maintained. Computational models have given researchers an efficient method by which to formulate hypotheses that can be tested experimentally. Discrete and hybrid models have been used to capture cell-level interactions. For example, a recent agent-based model of the normal morphogenesis of mammary epithelial acini explored the relative roles of apoptosis, proliferation, and apico-basal polarity in maintaining a physiologically normal epithelium. This model suggests that apoptosis is necessary and sufficient for lumen formation and that apico-basal polarity is required to establish the physiologically normal morphology of the epithelium [49]. A similar model examined the role of mammary progenitor cells in development of DCIS, and found that progenitor cells lead to greater genetic heterogeneity and faster formation of DCIS [63]. A recent study also explored tumor growth in a cylindrical domain and proposed a patient-specific model calibration protocol [51]. These and other models [47], [48] have provided valuable insight into the possible mechanisms underlying normal and abnormal development. With few exceptions [51], [64], most computational models of DCIS have focused on a spherical tissue or circular cross-section of a duct.

Here we established a 2D multi-cell lattice-based model of DCIS that incorporates cell proliferation, apoptosis, necrosis, adhesion, and contractility. All four morphologies (micropapillary, cribriform, solid and comedo) emerged in our model. High proliferation with low apoptosis led to the emergence of solid and comedo morphologies, low proliferation with high apoptosis led to the micropapillary morphology, and high proliferation with high apoptosis led to the cribriform morphology. Given that the morphology is established through a balance between proliferation and apoptosis, monitoring this in DCIS lesions could be a possible prognostic indicator of eventual progression to IDC. The parameters that led to the development of each morphology were similar qualitatively to those reported previously by others, with one notable exception: we found that the cribriform morphology resulted from cells dividing perpendicular to the epithelial layer, whereas a previously published model required the inclusion of an elevated pressure within the lumen of the duct, a so-called intraductal pressure [10]. We did not include intraductal pressure in our model since we could find little support for the existence of such a pressure in the literature. The citations discussed by Ref [10] in support of elevated levels of intraductal pressure in fact document increased interstitial fluid pressure (IFP). The presence of an IFP would impose forces directing inward on the epithelial duct, and not a force from the lumen that pushes outward as proposed by Ref [10]. In addition to suggesting regimes of parameters that lead to the four morphologies of DCIS, our model suggests probable transitions between these morphologies during breast cancer progression. Our model is unique in that, unlike most computational models of DCIS which examine cells arranged in a circular or spherical geometry, we also explored cell behavior in more physiologically relevant cylindrical and bifurcating duct geometries.

The results of our model are consistent with immunohistochemical studies that show high proliferation in comedo and solid morphologies compared to micropapillary and cribriform morphologies [58], [60], [65], [66]. As an example, Albonico et al found that 65% of cells in comedo lesions were positive for the proliferation marker Ki67, whereas only 3% of cells in cribriform lesions were Ki67-positive. Furthermore, we found that when proliferation was balanced by apoptosis, these lesions did not advance over time and remained either micropapillary or cribriform. Consistently, 100% of cells in cribriform lesions were found to express the apoptosis regulator Bcl-2, whereas this was reduced to 36% of cells in comedo lesions [65]. Our results are also consistent with clinical data showing that less than 50% of low-grade DCIS (lesions with a low proliferation rate) develop into invasive breast cancer over 25–30 years [67]. Similar to a recent computational study, we observed that increases in cell proliferation lead to the development of aberrant phenotypes and that disrupting proper cell division alignment can cause multiple lumena to form [68]. These results are congruent with those of a recent computational model that found that the ratio of tumor cell proliferation to apoptosis was a strong predictor of tumor volume [50], although this parameter does not correlate with histological grade. In the cylindrical and bifurcating duct geometries, the patterns that emerge in our model are consistent with our previously reported experimental results that show increased invasion from regions of high mechanical stress, more specifically from the ends of these tissues. The ability of cells to invade can be modulated by altering cell adhesion or contractility. Experimentally we have observed increased proliferation at the ends of these tissues. Interestingly our model showed increased invasion from the ends of tissues with and without preferential proliferation. This suggests that enhanced proliferation at the ends is not the cause of the invasion in these regions; experimental testing of this hypothesis would require the ability to spatially modulate cell proliferation, which is not yet possible. Importantly, we also observed that more than one morphology emerged simultaneously in these asymmetric tissue geometries, but not in the circular tissues. Different morphologies of DCIS have been frequently observed in histological sections of individual lesions [32], suggesting that future computational models of the mammary duct should incorporate more complex tissue geometries.

In order to accurately model the transition to invasive breast cancer in future simulations, it will be necessary to include loss of basement membrane integrity as a parameter. Furthermore, it would be beneficial to include extracellular matrix (ECM) regions to more rigorously incorporate cell-ECM interactions and explore the effect of heterogeneity in the ECM microenvironment on tumor cell invasion; early models that treat the ECM as a continuum have suggested an important role for crosstalk between the tumor cell and its surrounding stroma in tumor development [69], consistent with experimental results from mouse models of breast cancer [70], [71]. Here we assumed that cells become necrotic when they are 10 cell diameters away from the MEP layer. While this is a good average approximation based on clinical observations, it is important to note that cells do not always become necrotic at a given distance. Cells become hypoxic due to limitations in oxygen diffusion; however, ducts with diameters up to 500 µm have been observed without a necrotic core [42]. Furthermore, necrotic regions of tumors are heterogeneous and although apoptosis and necrosis are considered to be distinct modes of cell death, recent studies have suggested that they may lie on a continuum [72].

We focused on the morphology or architectural pattern of DCIS, which is characterized in the clinic using histology. It is important to note, however, that histological characterization (micropapillary, cribriform, solid, comedo) is not as accurate of a prognostic indicator of disease progression as classification systems that also take into account nuclear morphology or mitotic index (for example, the Nottingham [73] or Van Nuys [74] prognostic index). Ultimately, it would be beneficial to develop a multi-scale model of breast cancer that includes both cellular and subcellular features and behaviors. The mechanism by which cells and the ECM transmit mechanical cues and establish the mechanical profile of a tissue is incredibly complex [75]. To capture this complexity an ideal model would link lattice-based cellular behaviors with continuum biomechanical models and the subcellular machinery of the cytoskeleton to provide valuable insight into both normal and aberrant tissue behavior. Combining such computational models with recently developed engineered tumor models [76] may permit the successful integration of theoretical predictions with experimental validation. Our findings that tissue geometry-related mechanical stress plays a major role in the phenotypic evolution of DCIS point to the need to incorporate tissue structure information into individualized risk assessments, which could be accomplished with advances in high-resolution X-ray tomographic imaging.

## Supporting Information

S1 Figure **One-dimensional parameter of ratio of proliferation to apoptosis governs finally morphology.** (**A**) Varying the ratio of mitosis frequency divided by probability of apoptosis, we observe the emergence of solid and comedo morphologies at the highest ratio, and micropapillary morphology at the lowest ratio. (**B**) A similar trend is observed when cells can divide parallel to the outer layer of the duct or with a random orientation.
(TIF)
