# Supplementary figures and images for: Lattice-Based Model of Ductal Carcinoma In Situ Suggests Rules for Breast Cancer Progression to an Invasive State
Source: PLoS Comput Biol. 2014 Dec 4;10(12):e1003997. doi: 10.1371/journal.pcbi.1003997 (PMC4256017; doi:10.1371/journal.pcbi.1003997)

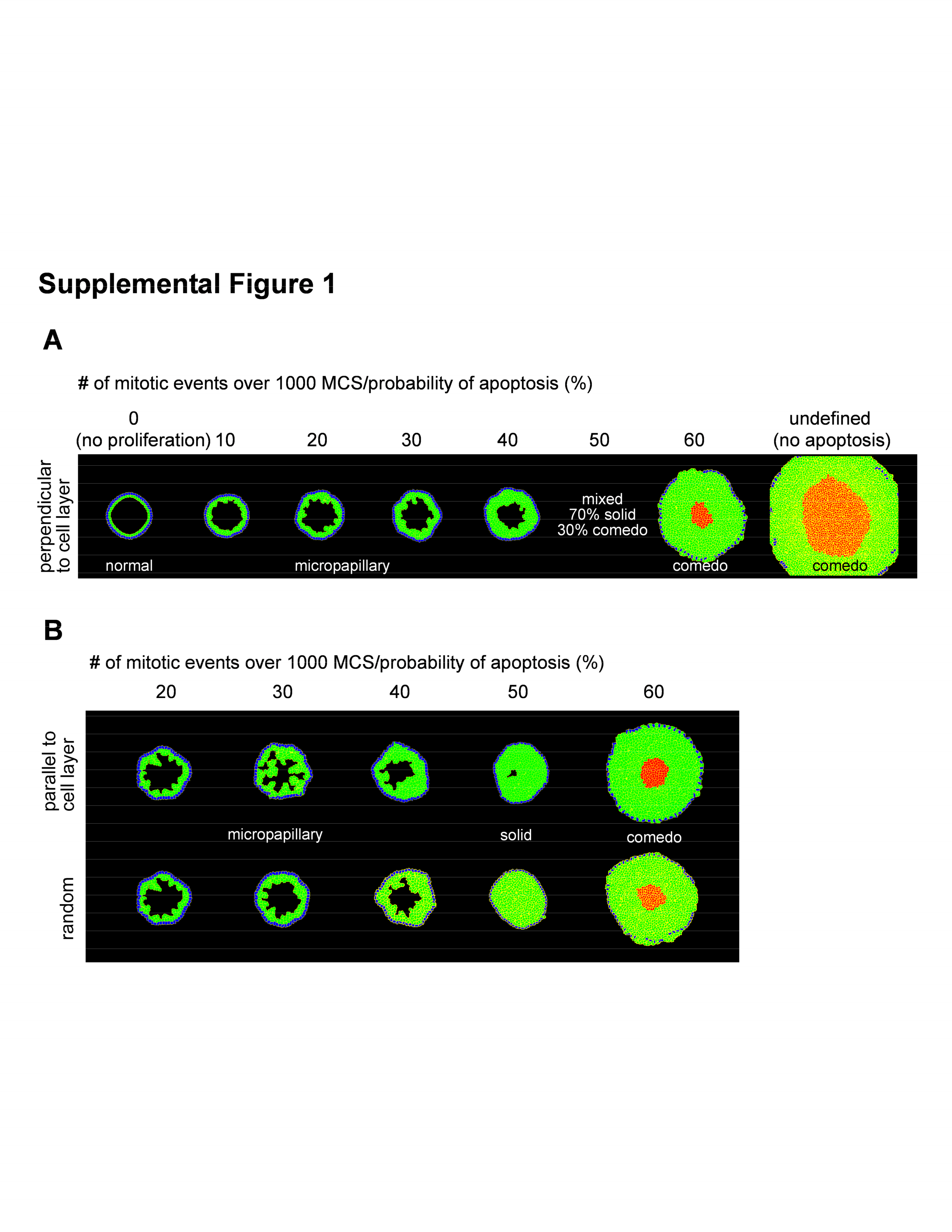

Supplement: S1 Figure — One-dimensional parameter of ratio of proliferation to apoptosis governs finally morphology. (A) Varying the ratio of mitosis frequency divided by probability of apoptosis, we observe the emergence of solid and comedo morphologies at the highest ratio, and micropapillary morphology at the lowest ratio. (B) A similar trend is observed when cells can divide parallel to the outer layer of the duct or with a random orientation. (TIF) [file pcbi.1003997.s001.tif]
